# Targeting PARP1: A Promising Approach for Next-Generation Poly (ADP-ribose) Polymerase Inhibitors

**DOI:** 10.1007/s12609-025-00582-5

**Published:** 2025-06-12

**Authors:** Alo Ray, Mateusz Opyrchal

**Affiliations:** Department of Internal Medicine, Indiana University School of Medicine, Indianapolis, IN USA

**Keywords:** PARP1, PARP2, DNA damage, Single-strand breaks (SSBs), Double-strand breaks (DSBs), Homologous recombination repair (HRR), Homologous recombination deficiency (HRD)

## Abstract

**Limitations of Poly (ADP‒ribose) Polymerase (PARP) Inhibitors:**

PARPis have demonstrated efficacy in BRCA-mutated cancers deficient in homologous recombination repair. Furthermore, PARPis have shown efficacy in BRCA-wild-type cancers with a homologous recombination deficiency phenotype known as BRCAness. Current clinically approved PARPis inhibit both PARP1 and PARP2, and their clinical promise is limited by toxicity, resistance, and a lack of combination partners.

**Recent Findings:**

PARP2 inhibition is associated with hematological toxicity, affecting the tolerability and efficacy of monotherapy and combination therapies. Furthermore, synthetic lethality in BRCA-mutated cancers depends mostly on PARP1, whereas PARP2 is not essential. These findings promoted the development of next-generation PARPis with greater selectivity for PARP1 than for PARP2.

**Summary:**

In this review, we discuss the next-generation PARPis that target PARP1 and show promise in terms of improved safety, tolerability, pharmacological profiles, and efficacy compared to existing clinically approved PARPis. These next-generation PARP1-selective inhibitors hold significant promises for improving the survival and outcomes of cancer patients.

## Introduction

Poly (ADP‒ribose) polymerase (PARP) plays a pivotal role in cellular physiology by catalyzing the transfer of ADP‒ribose from nicotinamide (NAD +) precursors to target proteins, a posttranslational modification called PARylation [1, 2]. This enzymatic process, termed poly (ADP)-ribosylation, regulates vital cellular pathways [2, 3]. PARP1 and PARP2 are the best characterized PARPs (which refers to PARP 1 and 2) and contribute to most PARP activities [4–6]. PARP1 and PARP2 are well characterized for their role in DNA damage repair, ensuring cell survival by modifying proteins involved in chromatin architecture and DNA repair processes [5, 7, 8]. PARP1 has the predominant role in DNA damage repair and PARP2 has a lesser effect [9]. PARP is catalytically activated by single-strand breaks (SSBs) and double-strand breaks (DSBs) [5, 10–13]. Subsequently, PARP promotes the formation of poly (ADP‒ribose) (PAR) chains, which are important for providing a docking platform for DNA repair proteins to damaged sites, facilitating their recruitment [1, 10, 12]. Finally, the PAR chains are removed from substrate proteins by dePARylating enzymes [14, 15]. Recently, a variety of ADP-ribose degrading enzymes have been identified with different substrate specificities. Poly (ADP-ribose) glycohydrolase (PARG) is the major dePARylating enzyme which hydrolyzes the glycosidic linkages between ADP-ribose units of PAR polymers to generate free ADP-ribose monomers [14, 15]. Recently, other dePARylating enzymes such as ADP-ribose hydrolases, phosphodiester ADP-ribose hydrolases, macrodomain-ADP-ribose erasers, have been identified which are also involved in removing PAR chains (reviewed in [16]. Owing to the catalytic function of PARP, PARP can tightly bind to DNA, resulting in “PARP-DNA” trapping, which is stabilized by PARP inhibitors (PARPis), blocking the replication machinery and facilitating the collapse of the replication fork [17]. Disruption of these processes by pharmacological inhibition has emerged as a promising strategy in cancer therapy.

### PARP Plays a Role in Base Excision Repair/Single-Strand Break Repair and DNA Replication

**Fig. 1:**
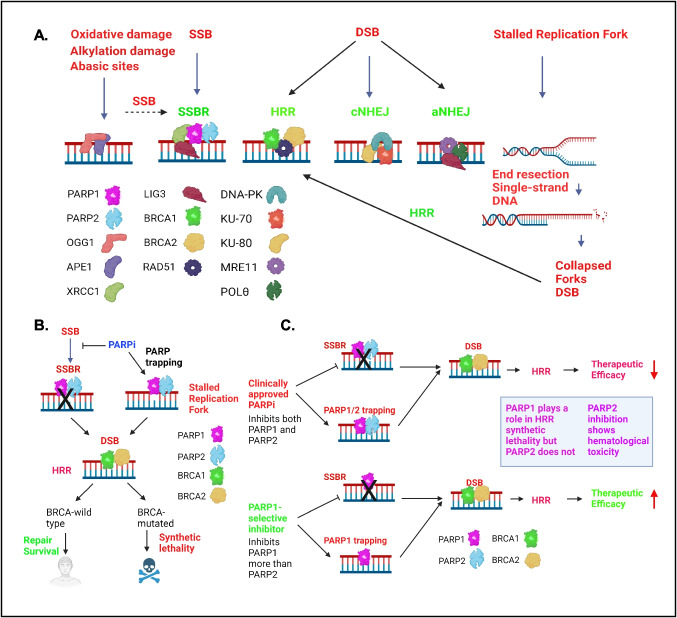
Major cellular DNA damage repair pathways related to PARP function and the mechanism of synthetic lethality between PARP inhibitors and HRR deficiency.** A**. PARP1/2 predominantly play a role in SSBR. Inhibition of SSBR results in DSBs. PARP inhibition and PARP trapping result in stalled replication forks, which are converted to DSBs. In the presence of BRCA1/2, DSBs are predominantly repaired by HRR. In the absence of BRCA1/2, cells repair DSBs via the cNHEJ and aNHEJ pathways. BER (base excision repair), SSBR (single-strand break repair), HRR (homologous recombination repair), cNHEJ (classical nonhomologous end joining), aNHEJ (alternative nonhomologous end joining). **B**. Schematic showing the synthetic lethality between a PARPi and HRR-deficiency. **C**. Schematic showing how PARP1-selective inhibitors improve PARP efficacy. PARP1 is needed for the synthetic lethality of HRR and PARP1 selective inhibitors limit toxicity

Due to spontaneous DNA damage, oxidative stress, irradiation, and reactive endogenous metabolites, thousands of DNA lesions are introduced in the DNA every day. These DNA modifications are repaired by base excision repair (BER) and single-strand break repair (SSBR). BER is the major pathway for repairing oxidative base damage, alkylation damage, and abasic sites [18]. In BER, damage is detected by OGG1 and APE1, which create SSBs that are repaired by the SSBR pathway [5, 13] (Fig. 1A). PARP1/2 binds to SSBs with high affinity and becomes activated, inducing ADP-ribosylation of histones and other DNA repair proteins and essential for BER/SSBR pathways [5, 19]. The recruitment of the core factor XRCC1 to SSBs is dependent on PARP1 and PARP2 [20]. XRCC1 functions as a scaffold for DNA Polβ, LIG1/3 and PNKP needed for the repair process [5, 18] (Fig. 1). Thus, BER/SSBR restores the damaged DNA to intact DNA [8].

PARP1 interacts and stimulates the activity of multiple replication proteins and influences DNA replication process [21–24]. Replication stress caused by oncogene activation, dNTP depletion, and unrepaired SSBs. These SSBs encounter the replication machinery resulting in stalled and/or collapsed replication fork with a single-ended DNA DSBs [8]. PARP1 has been implicated in protecting the degradation of stalled DNA replication forks and DSBs by promoting the recruitment of MRE11 and RAD51 [5, 25, 26]. These DSBs are resolved by the homologous recombination repair (HRR) pathway (described further below). PARP1 also plays a role in the prevention of untimely restart of stalled replication forks by binding to RECQ1 and allowing repair of DNA lesions [5]. Recently, PARP1 has been implicated to play a direct role in the Okazaki fragment processing [27, 28]. These studies showed that the unligated Okazaki fragments is one of the major sources of PARP activity and perturbation of the DNA replication proteins LIG1 or FEN1 increases the S phase PARP independent of damaged DNA or replication stress [27, 28].

### PARP Inhibitors Inhibit Single-Strand Break Repair, DNA Replication, and induce PARP Trapping Resulting in Double-Strand Breaks which are Repaired by Homologous Recombination Repair Pathway

PARPis catalytically inhibit PARP1/2, and the Inhibition of SSBR by PARPi results in accumulation of SSBs. When these SSBs are unrepaired and enter the S phase, they are converted to more lethal DSBs. Furthermore, upon PARPi treatment, PARP binding to the DNA breaks is stabilized resulting in PARP trapping (Fig. 1A) [17, 29]. These “trapped-PARPs” causes replication fork stalling and SSBs which eventually gets converted to DSBs (Fig. 1A) [17, 30–33] (Fig. 1A). Studies supported that trapped PARPs form a complex with damaged DNA which is more cytotoxic compared with unrepaired SSBs due to PARP inhibition [17]. In cells harboring wild-type BRCA1/2 genes, these DSBs are mainly repaired via the HRR pathway, an error-free repair pathway which predominantly works in S phase and to a lesser extent in G2 phase of cell cycle due to availability of the homologous chromatid for recombination (Fig. 1A) [8, 34–36]. In the canonical HRR, BRCA2 initially binds to RAD51 and localizes to the DSB sites, whereas BRCA1 plays a crucial role in DSB resection and signal transduction [37, 38]. BRCA1 interacts with PALB2 and helps localization of BRCA2 by forming a molecular scaffold of BRCA1-PALB2-BRCA2 needed for HRR [39]. In the absence of functional BRCA1 and BRCA2, DSBs are resolved through the error-prone classical nonhomologous end joining (cNHEJ) pathway, or the alternative nonhomologous end joining (aNHEJ) pathway (Fig. 1A). During cNHEJ process, DNA-PKcs, KU-70, and KU80 bind the DSBs. 53BP1 limits end resection to create an overhang to promote ligation by DNA ligase 4 and XRCC4-XLF [5]. During the aNHEJ, DSBs use microhomologies to align the broken DNA ends. MRE11, POLꝊ and other proteins bind to the microhomology region and LIG3 play a role in the ligation of broken DNA ends (Fig. 1A) [8, 32, 40]. The ligation of broken DNA ends often lead to genomic alterations and increased heterogeneity in the genome [8, 32, 36, 40].

### PARP Inhibitors Exhibit Synthetic Lethality in BRCA-Mutated Cancers

As described in the previous section, the unrepaired SSBs, defective replication, and PARP trapping induce replication stress-induced DSBs. BRCA-wild-type cells repair these DSBs via the HRR pathway [37, 38], but the BRCA-mutated cells cannot repair these DSBs. Therefore, inhibition of PARP in BRCA-mutated cells causes synthetic lethality due to the presence of unrepaired DSBs [41–46] (Fig. 1B). As a result, BRCA1/2-mutated cells are hyper reliant on PARP-mediated DNA repair for their survival [31, 41, 42]. This laid the foundation for the development of targeted therapies by exploiting the vulnerability of BRCA1/2-mutated tumors to homologous recombination deficiency (HRD) [47]. Currently, HRD is used as a predictive biomarker for PARP treatment and several methods are being used to determine the level of HRD in cancers [8].

### PARP Inhibitors Exhibit Synthetic Lethality in Patients with the Brcaness Phenotype

Numerous investigations have shown that in addition to classical BRCA1 and BRCA2 mutations, a diverse array of molecular alterations can contribute to the development of HRD [40, 48–50]. Defects in these non-BRCA mutated proteins can promote a"BRCAness"phenotype, characterized by biological and clinical features akin to those associated with BRCA1/2 mutations [40, 48–50]. These alterations include mutations in key genes that play a role in HRR, such as RAD50, RAD51, RAD51C, RAD51D, RAD54L, and RAD51C58, as well as the overexpression of the HORMAD1 protein, which suppresses RAD51-dependent HRR [48, 49, 51, 52]. Additionally, defects in DNA damage response genes associated with HRR, such as BARD1, BRIP1, ATM, ATR, CHK1, CHK2, NBS1, PRKDC, RPA1, and DSS1, and mutations in several Fanconi anemia pathway genes, including PALB2, FANCA, FANCC, FANCD2, FANCE, and FANCF, have been implicated in the development of HRD [46, 48, 53]. Moreover, mutations in non-HRR DNA damage response genes such as loss of CDK12 can induce genomic instability resembling BRCAness across various cancer types, indirectly promoting HRR deficiency [54, 55]. CRISPR-Cas9 loss-of-function screens in PARPi-insensitive cell lines has identified most frequently inactivated genes in tumors and showed that XRCC1 is a potential new prognostic biomarker of PARPi sensitivity in prostate cancer [56].

Other mechanisms, including promoter methylation, somatic BRCA1/2 mutation, and gene deletion, can also impair BRCA1/2 function, resulting in a BRCAness phenotype [57–59]. PARPi induce synthetic lethality not only in patients harboring hereditary BRCA1/2 mutations but also in those exhibiting the BRCAness phenotype and HRD [41, 54]. Considering all these factors, HRR gene mutations beyond BRCA1/2 and overall dysregulation of HDR can serve as biomarkers to predict sensitivity to PARPis, thereby guiding personalized therapeutic strategies [48].

### First-Generation PARP Inhibitors Targeting both PARP1 and PARP2

Multiple PARPis have undergone successful clinical trials and have been approved by the Food and Drug Administration for ovarian, breast, pancreatic, and prostate cancers, demonstrating the promise of this approach [31, 60, 61]. These PARPis exhibit varying potencies as both PARP catalytic function inhibitors and PARP trapping agents and have been reviewed elsewhere [17, 31, 62, 63]. In general, the first-generation PARPis olaparib, talazoparib, niraparib, rucaparib, veliparib, pamiparib, and fuzuloparib target both PARP1 and PARP2, bind to their active sites and inhibit PARylation. This is because PARP1 and PARP2 share a high degree of sequence homology (69%) at the catalytic domain [64]. Even though all these inhibitors trap PARP at sites of DNA damage they show varied efficacy in PARP trapping [30, 61]. Compared with other agents, talazoparib results in the greatest amount of PARP trapping, in contrast with veliparib, which has weaker PARP trapping activity [30]. Several clinical trials have extensively explored the clinical efficacy of olaparib [65], talazoparib [66, 67], niraparib [68], rucaparib [69], veliparib [70], pamiparib [71], and fuzuloparib [72] in patients harboring BRCA mutations. These first-generation PARPis demonstrate distinct levels of synthetic lethality with HRD tumors and various levels of toxicity due to their differences in PARP selectivity and trapping ability [17, 31, 53, 62, 63, 73]. Specifically, PARPis showed dose limiting toxicities when used in combination with chemosensitizers and radiosensitizers [8, 74]. In addition, while above PARPis have demonstrated promising efficacy, not all patients respond to these treatments, especially BRCA-proficient patients (reviewed in [35, 60, 75]. Furthermore, PARPi efficacy is often diminished by the emergence of drug resistance [60, 76]. Several mechanisms have been demonstrated for PARPi resistance, such as drug efflux [13, 77, 78], decreased PARP trapping to DNA [78, 79], and the restoration of HRR through various mechanisms (reviewed elsewhere), such as BRCA mutation reversion and PARP1 mutation [60, 76, 78, 80–82], increased BRCA and other HRR protein expression [60], the stabilization of the replication fork [13, 76, 78, 83], the inactivation of different NHEJ-promoting factors that inhibit DNA end resection [76], and other processes, such as the loss of *POLQ* [78], as well as the overexpression of *CDK12* and *WEE1* [49, 78]. Secondary mutations in BRCA1/2, RAD51 C, RAD51D, and PALB2 are common which restores the functional protein have been reported in many cancer models including breast, ovarian, pancreatic, and prostate cancers (reviewed in [84]. Genome-wide and high-density CRISPER-Cas9 screens identified PARP1 point mutations in the DNA binding site and outside the DNA binding site causing PARPi resistance by lowering PARP trapping [81, 85]. PARP1 mutations p.R591 C and p.848 delY, were identified to cause PARPi resistance. Specifically, the p.R591 C, a clinically relevant PARP mutation found in an olaparib-resistant ovarian cancer patients promotes dissociation of PARP1 from the DNA damage site showing inefficient PARP trapping resulting in PARP resistance [81].

### Why the Development of Next-Generation PARP Inhibitors is Important for Improving PARP Inhibitor Efficacy

The first-generation PARPis bind with PARP1 and PARP2 showing similar levels of inhibition [64]. These PARPis generally shows adverse events, including hematological effects, gastrointestinal effects, renal toxicities, fatigue etc. [86–88]. Emerging evidence suggests that the inhibition of PARP2 results in notable hematological toxicity due to the critical role of PARP2 in the survival of hematopoietic stem cells [89, 90]. In preclinical mouse models, the loss of both PARP1 and PARP2 is incompatible with normal embryonic development, whereas the deletion of PARP2, but not PARP1, leads to chronic anemia, supporting a role of PARP2 in erythropoiesis [90]. PARP2 knockout mice exhibit impaired differentiation of erythroid progenitor cells and reduced erythrocytic lifespan, thymopoiesis, adipogenesis, and spermatogenesis, which are correlated with the toxicity of first-generation PARPis [89–93]. The hematologic toxicities limit the use of current clinically approved PARPis as monotherapies, especially in a relapsed setting, and in combination therapy treatments [91, 92]. Another important factor, for developing PARP1 selective inhibitor is that PARP1 plays a major role in 80–90% of PARylation induced by DNA damage, whereas PARP2 accounts for only 5–20% of PARylation [94]. As a result, the synthetic lethality of PARPis in HRR-deficient tumors primarily depends on PARP1 inhibition and trapping, while PARP2 inhibition and trapping are not essential [6, 17]. Therefore, it has been hypothesized that selectively inhibiting PARP1 would reduce PARPi toxicity associated with PARP2 inhibition while improving synthetic lethality in HRD cancers and subsequently improving the efficacy of PARPi treatments. These factors led to the development of next-generation PARPis with greater selectivity for PARP1 than for PARP2.

### Next-Generation PARPis Demonstrate Greater PARP1 Selectivity than PARP2 Selectivity and Display Greater PARP Inhibitor Efficacy

The effort to identify PARP1 selective inhibitor led to the first discovery of 2-[1-(4,4-Difluorocyclohexyl)piperidin-4-yl]−6-fluoro-3-oxo-2,3-dihydro-1H-isoindole-4-carboxamide (NMS-P118): A potent, orally available, and highly selective PARP1i with excellent absorption, distribution, metabolism, and excretion (ADME) and pharmacokinetic (PK) profile showing high efficacy in breast cancer and pancreatic cancer xenograft models [95]. Following this discovery, many laboratories tried to develop PARP1 selective inhibitors and some of them are in clinical trial and others are in pre-clinical development.

### Next-generation PARP1 Selective PARPis in Clinical Development

Several next-generation PARPis designed with higher PARP1 selectivity than PARP2 selectivity are currently in clinical trials (Table 1). AZD5305 (saruparib), a highly potent and selective PARP1i (reviewed in [93]), exhibited 500-fold selectivity for PARP1 over PARP2 in a PARylation assay (IC_50_ of 1.55 nM for PARP1 and IC_50_ of 653 nM for PARP2) [96, 97] and showed excellent PARP1 trapping capacity compared with that of PARP2 [96]. AZD5305 also exhibited greater PARylation (IC_50_ of 2.3 nM) than talazoparib (IC_50_ of 5.1 nM) and veliparib (IC_50_ of 33 nM) did [96]. AZD5305 had a weaker effect on hematopoietic stem/progenitor cells than talazoparib did [97] and had minimal effects on hematological parameters compared to olaparib and niraparib in rat preclinical models [96]. AZD5305 displayed excellent safety and tolerability profiles and favorable pharmacokinetic (PK) and pharmacodynamic (PD) properties in BRCA-mutated tumor models [96]. Mechanistically, AZD5305 induces the accumulation of DNA damage and G2/M cell cycle arrest in HRR-deficient colon, breast, and gastric cancer cell lines [96]. Compared with other PARPis, AZD5305 has better efficacy and improved overall and progression-free survival in HRR-deficient breast, ovarian, gastric, and colon cancer cell lines; breast, ovarian, pancreatic, and colon cancer cell-derived xenograft (CDX) models; and breast cancer patient-derived xenograft (PDX) models [96, 98, 99]. AZD5305 also resulted in significant tumor regression in combination with carboplatin and paclitaxel in BRCA-wild-type breast cancer CDX models and improved tolerability in combination with carboplatin compared with first-generation PARPis in both breast cancer CDX and breast and ovarian cancer PDX models [96, 98]. The phase I/IIa clinical trial PETRA study (NCT04644068,www.clinicaltrial.gov, Table 1) evaluated the safety and early clinical efficacy of AZD5305 as monotherapy or in combination with other anticancer agents (paclitaxel, carboplatin with or without paclitaxel) in advanced solid cancers. Trial results with HRR-deficient (with mutations in one of five HRR genes: BRCA1, BRCA2, PALB2, RAD51C, or RAD51D) breast, ovarian, pancreatic, and prostate cancer patients revealed that, compared with first-generation PARPis, AZD5305 exhibited excellent safety and tolerability profiles and favorable PK and PD properties with a wide therapeutic index [100]. AZD5305 inhibited approximately 90% of PARP activity in tumor tissue collected from biopsies of these patients [100]. Efficacy has been observed in all tumor types across all doses, and mutation types showing great promise compared with clinically approved PARPis [100]. The efficacy and safety of AZD5305 in combination with new hormonal agents (enzalutamide, abiraterone, acetate, and darolutamide) are being assessed in a phase I/IIa PETRANHA study (NCT05367440, www.clinicaltrial.gov, Table 1) of metastatic castration-sensitive and castration-resistant prostate cancers [101]. In addition, the efficacy and safety of AZD5305 in combination with a physician’s choice of new hormonal agents (abiraterone, darolutamide, or orenzalutamide) compared with those of the placebo are being evaluated in a phase III EvoPAR-Prostate01 study with metastatic castration-sensitive prostate cancer (NCT06120491, www.clinicaltrial.gov, Table 1) [102]. Another clinical trial has been initiated in newly diagnosed prostate cancer patients, where AZD5305 will be evaluated alone and in combination with darolutamide, an antiandrogen drug used to treat nonmetastatic castration-resistant prostate cancer in men (NCT05938270, www.clinicaltrial.gov, Table 1). AZD5305 is also in clinical trials in combination with camizestrant, an oral selective estrogen receptor degrader, and will be compared with CDK4/6 inhibitors plus endocrine therapy or with camizestrant in HR-positive, Her2-negative, BRCA1-, BRCA2-, or PALB2-mutated advanced breast cancer (NCT06380751; www.clinicaltrial.gov, Table 1). AZD5305 has also been investigated in combination with datopotamab deruxtecan (Dato-DXd). Dato-DXd is a TROP2-directed antibody drug conjugate composed of a topoisomerase I inhibitor (an exatecan derivative, DXd) conjugated to datopotamab, a humanized anti-TROP2 IgG1 antibody, via a linker. Topoisomerase I inhibitors induce SSBs that require PARPs for their repair. The AZD5305 and Dato-DXd combination enhanced the sensitization of triple-negative breast cancer (TNBC) and non-small cell lung cancer cell lines and resulted in superior tumor growth inhibition in a gastric cancer CDX and a PARPi-resistant ovarian cancer PDX model. This combination is being investigated in clinical trials in patients with advanced/metastatic solid tumors in the TROPION-PanTumor03 study (NCT05489211, www.clinicaltrial.gov, Table 1) [103]. AZD5305 also exhibited promising efficacy with an ATR inhibitor (ATRi) in PARPi- and ATRi-resistant HRR-proficient- and -deficient cell lines, in breast and ovarian cancer CDX, and in TNBC and ER + breast cancer PDX models [104]. Currently, a clinical trial evaluating the AZD5305 and ATRi (ceralasertib) combination (NCT02264678, www.clinicaltrial.gov, Table 1) in advanced solid tumors is ongoing.

**Table 1:** Next-generation PARP inhibitors in clinical trials showing increased PARP1 selectivity over PARP2. PARP1 selectivity over PARP2 was measured via an enzymatic/PARylation assay, and DNA trapping was measured via a DNA trapping assay. The clinical trial data are from www.clinicaltrial.gov. mCSPC (metastatic castration-sensitive prostate cancer), mCRPC (metastatic castration-resistant prostate cancer), HRR (homologous recombination repair), HRD (homologous recombination deficiency), IDH (isocitrate dehydrogenase), GBM (glioblastoma)

PARP inhibitor	PARP selectivity over PARP2	PARP1-DNA trapping over PARP2	Clinical trial	Phase of development (two places)	Cancer type	Key eligibility criteria	Developed by (Company/Institution)
**AZD5305** **AZD5305 + Paclitaxel** **AZD5305 + ** **Carboplatin (with or without Paclitaxel)**	500-fold	> PARP2	NCT04644068	Phase I/IIa	Solid tumors	Prior PARPi treatment required	AstraZeneca, Cambridge, United Kingdom
**AZD5305 + new hormonal agents**			NCT05367440	Phase I/IIa	Prostate (mCSPC and mCRPC)	no prior PARPi treatment	
**AZD5305 + physicians’ choice new hormonal agents**			NCT06120491	Phase III	Prostate (mCSPC)	Confirmed HRRm status	
**AZD5305** **AZD5305 + Darolu-tamide**			NCT05938270	Phase I (to be open)	Prostate	no HRR status requirement published	
**AZD5305** ** + Camizestrant**			NCT06380751	Phase III	Breast	BRCA1, BRCA2, or PALB2 Mutations	
**AZD5305 + Dato-Dxd**			NCT05489211	Phase II	Solid tumors	no HRR status requirement published	
**AZD5305 + Ceralasertib**			NCT02264678	Phase I/Ib	Solid tumors	known or suspected BRCA mutation, PALB2 mutation, RAD51 C/D mutation or HRD positive status	
**NMS-03305293**	> 200-fold	No trapping activity	NCT04182516	Phase I	Solid tumors	BRCA1 and BRCA2 mutation status is not required for enrollment in the dose escalation part, but enrichment with deleterious/pathogenic or likely pathogenic/suspected deleterious BRCA carriers will be attempted	Nerviano Medical Sciences, Italy
**NMS-03305293 + TMZ**			NCT04910022	Phase I	Diffused Glioma	no HRR status requirement published	
**NMS-03305293 + TMZ**			NCT04910022	Phase II	IDH wild type GBM	IDH wild type no HRR status requirement published	
**AZD9574** **AZD9574 + targeted agents**	> 20-fold	> PARP2	NCT05417594	Phase I/IIa	Solid tumors	deemed suitable for a Poly ADP-Ribose Polymerase (PARPi) by the Investigator	AstraZeneca United Kingdom
**HRS-1167**	> PARP2	Not known	NCT05473624	Phase I	Solid tumors	HRR gene mutation	Jiangsu HengRui Medicine Co., Ltd., China
**IMP1734**	PARP1 selective	Not known	NCT06253130	Phase I/II	breast, ovarian, mCRPC	deleterious or suspected deleterious germline or somatic mutations of select HRR genes	Eikon Therapeutics, United States

NMS-03305293 (NMS-P293) is an oral, PARP1-selective inhibitor showing > 200-fold selectivity for PARP1 over PARP2 in the PARylation assay, but this compound is not a good PARP-trapper [105–107], (Table 1). Owing to its lower inhibitory effect on PARP2, NMS-03305293 spares normal myelocytes [105]. NMS-03305293 exhibited a good PK profile and good ADME properties, including a low efflux ratio and high cross-species metabolic stability in rodents and nonrodent models [105]. NMS-03305293 exhibited good efficacy in BRCA-mutated breast cancer cell lines and CDX models [105, 106]. A phase I dose-escalation and dose expansion study explored the safety, tolerability, and antitumor activity of NMS-03305293 as a single agent in patients with advanced/metastatic, relapsed/refractory solid tumors for whom exhausted standard treatment options or standard therapy are unavailable (NCT04182516, www.clinicaltrial.gov, Table 1). In addition, NMS-03305293 exhibited high blood‒brain barrier (BBB) penetration and represents a novel therapeutic option for glioblastoma (GBM) and localized brain metastasis [106]. On the basis of this property, the PARPA-293–002 study evaluated the safety and efficacy of the NMS-03305293 and temozolomide (TMZ) combination in adult diffuse glioma patients (phase I) and in isocitrate dehydrogenase (IDH) wild-type GBM patients (Phase II) at first relapse (NCT04910022, www.clinicaltrial.gov, Table 1). NMS-03305293 was well tolerated in this clinical trial, with no dose-dependent trend toward myelosuppression and demonstrated encouraging efficacy in combination with TMZ [108].

AZD9547 showed greater PARP1 binding in a fluorescence anisotropy assay, with > 8000-fold greater PARP1 binding than PARP2 binding. AZD9547 is more effective at promoting PARylation in PARP2-/- cells (IC_50_ of 1.5 nM) than in PARP1-/- cells (IC_50_ > 30 nM), with ~ 20-fold greater selectivity for PARP1 than for PARP2 in the enzymatic assay. Furthermore, AZD9574 is more potent at PARylation (IC_50_ of 1.5 nM) than olaparib is (IC_50_ of 14.7 nM), with ~ 9.8-fold greater PARylation than olaparib in the enzymatic assay [109]. Notably, compared with the first-generation PARPi talazoparib, olaparib, or pamiparib, which trap both PARP1 and PARP2, AZD9547 showed only PARP1 trapping to DNA and no PARP2 trapping. Consequently, compared with olaparib and pamiparib, AZD9547 demonstrated decreased hematologic toxicity in rat preclinical models and displayed favorable PK and PD properties [109]. AZD9574 demonstrated robust anticancer efficacy as a monotherapy in HRR-deficient breast, ovarian, and colon cancer cell lines and in a breast cancer CDX model [109, 110]. Owing to its ability to increase the BBB, AZD9574 showed improved efficacy and extended survival in combination with TMZ in a GBM model [109]. Currently, AZD9574 is in clinical trials as monotherapy and in combination with other anticancer agents for advanced solid tumors (both HRR-proficient and HRR-deficient) that have been used to assess its safety, tolerability, PK, and PD (NCT05417594, www.clinicaltrial.gov; Table 1). This clinical trial will include advanced/relapsed ovarian, breast, pancreatic, and prostate cancer patients who can be treated with PARPis and HER2-negative breast cancer patients with BRCA1/2, PALB2, RAD51C, and RAD51D mutations. This clinical trial will also assess the combination of AZD9574 with trastuzumab deruxtecan in HER2-positive advanced metastatic solid tumors, Dato-DXd in advanced metastatic solid tumors, and TMZ in glioma patients with IDH mutations. 

The safety, tolerability, and efficacy of the PARPi HRS-1167 (M9466), a highly selective PARP1i, were assessed in a phase I clinical trial in advanced solid tumors (NCT05473624, www.clinicaltrial.gov, Table 1). Patients with advanced solid tumors that progressed on standard therapies or for whom no standard therapies were available were eligible for the dose escalation study. On the other hand, previously treated patients with germline or somatic BRCA1/2, PALB2, or RAD51 mutations received this drug in the dose expansion cohort. In the clinical trial, no dose-limiting toxicities occurred, and the maximum tolerated dose was not reached. Additionally, HRS-1167 exhibited favorable PK and safety profiles in these patients, indicating promising antitumor efficacy [111].

IMP1734, a selective PARP1i, reduces tumor growth in preclinical models. It is currently being evaluated in clinical trials in patients with advanced, recurrent, and metastatic breast, ovarian, and prostate cancers with mutations in select HRR genes. The primary endpoint is safety and tolerability, and the secondary endpoint is efficacy, progression-free survival, and overall survival. The exploratory endpoints include PK, PD, and patients’ quality of life (NCT06253130, www.clinicaltrial.gov, Table 1) [112].

### Preclinical Development of Next-Generation PARP1-Selective PARPis

Several PARPis showing greater selectivity for PARP1 than for PARP2 have shown promising results in preclinical studies.

VB15010 is a novel and potent selective PARP1i (Table 2). VB15010 exhibited greater selectivity for PARP1 than other PARP family members did (30– to 8,000-fold) in enzymatic assays and superior selectivity for PARP1 over PARP2 DNA trapping (> 1700-fold) in DNA trapping assays. VB15010 demonstrated good PK and PD properties and potent antiproliferative activity in both BRCA-mutated and HRD-positive cancer cell lines and demonstrated substantial antitumor activity in BRCA-mutated CDX and HRD-positive PDX models. VB15010 showed excellent bioavailability in animal models and displayed preferable tumor tissue distribution. Overall, higher PARP1 selectivity over PARP2, durable DNA trapping capability, improved PK and PD properties, favorable tumor‒to‒plasma distribution ratios, and potent antitumor activity show promise for enhancing the clinical efficacy of VB15010 and reducing hematological toxicity in patients [113].

**Table 2:** Next-generation PARP inhibitors in preclinical stages showing increased PARP1 selectivity over PARP2. PARP1 selectivity over PARP2 was measured via an enzymatic/PARylation assay, and DNA trapping was measured via a DNA trapping assay. TNBC (triple-negative breast cancer)

PARP inhibitor	PARP1-selectivity over PARP2	PARP1-DNA trapping over PARP2	Cell lines/CDX/PDX	Developed by (Company/Institute)
VB15010	30–8000 fold	1700-fold	HRR-positive cancers	Shenzhen Yangli Pharmaceutical Technology, China
D0112-005	2000-fold	66-fold	Breast	Chengdu Easton Biopharmaceuticals Co., Ltd., Chengdu, China
XZP-7797	> 1000-fold	Not known	Breast, pancreatic	Sihuan Pharmaceutical, China
SNV001	> 500-fold	> PARP2	Breast	Synnovation Therapeutics, United States
ACE86225106	72-fold	131-fold	Solid tumors	Acerand Therapeutics, China and United States
DM5167	6.88-fold	348-fold	TNBC	Kainos Medicine, Inc., Republic of Korea
HH102007	> AZD5305 and AZD9574	> AZD9574	Breast, pancreatic	Haihe Biopharma, China
HSK40495	> PARP2	Not known	Breast, colorectal	Haisco Pharmaceutical, China
DSB1559	> AZD5305	Not known	TNBC	Duke Street Bio Ltd, United Kingdom
LAE119	PARP1 selective	> 1000-fold	Breast, pancreatic, colorectal	Laekna Pharmaceuticals, China
DHC-1	PARP1 selective	Not known	Breast, pancreatic	Qingdao University of Science and Technology, China

Another PARP1-selective inhibitor, D0112-005, showed 2000-fold selectivity for PARP1 over PARP2 and PARP3 and 66-fold greater PARP1 trapping than PARP2 (Table 2). DO112-005 exhibited a good safety profile in rats and dogs and showed high anti-proliferative activity against BRCA-mutated breast cancer cell lines and CDX models [114].

XZP-7797 is a potent, selective, and CNS-penetrating PARP1-selective inhibitor that shows more than 1000-fold selectivity for PARP1 over PARP2 and other members of the PARP family in PARylation assays and has good ability to trap PARP1 (Table 2). Accordingly, XZP-7797 did not have any adverse hematological effects in a toxicity study using rat model. XZP-7797 also showed good PK and ADME properties and good BBB penetration in a mouse model and demonstrated significant efficacy in breast cancer cell lines, a BRCA1-mutated breast cancer CDX model, and a pancreatic cancer CDX model [115].

SNV001 demonstrated > 500-fold PARP1 selectivity over PARP2 in the PARylation assay and trapped PARP1 on DNA but not PARP2, resulting in less toxicity in mouse models [116] (Table 2). Compared with olaparib, SNV001 dose-dependently inhibited HRD cancer cell lines, including breast and colorectal cancer, and inhibited tumor growth in a BRCA-mutated breast cancer CDX model without any signs of toxicity [116].

ACE86225106, another PARP1-selective inhibitor, showed 72-fold greater PARP1 selectivity over PARP2 in an enzymatic assay and 131-fold greater PARP1 trapping over PARP2 in a DNA-trapping assay (Table 2). ACE86225106 exhibited lower inhibition of hematopoietic stem cells, a good safety profile, and a unique PK profile, with a long half-life and low distribution volume. Consequently, ACE86225106 exhibited robust efficacy in cancer cell lines and CDX models, indicating good clinical utility [117].

DM5167 is a PARP1-selective inhibitor that shows 6.88-fold greater PARP1 selectivity over PARP2 in an enzymatic assay and 348-fold greater PARP trapping activity in a DNA trapping assay (Table 2). Compared with olaparib, DM5167 showed acceptable safety margins and toxicity profiles and demonstrated good anticancer efficacy in TNBC cell lines and CDX models [118].

HH102007, a highly potent PARP1-selective inhibitor, studied extensively and compared with AZ5305 and AZD9574 compounds. HH102007 exhibited greater PARP1 selectivity than did AZD5305 and AZD9574 in enzymatic assays and showed lower hematological toxicity than these compounds in a rat model. However, HH102007 traps more PARP1 than AZD9574 (Table 2). Additionally, HH102007 also exhibited high potency in BRCA-mutated breast cancer cell lines and CDX models alone and in combination with carboplatin, showing greater efficacy than did AZD9574 [119].

HSK40495, a PARP1-selective inhibitor, showed greater PARP1 selectivity than PARP2 in an enzymatic assay and 5000-fold greater selectivity than PARP2 in a protein binding assay (Table 2). Compared with talazoparib, HSK4095 was less cytotoxic to bone marrow progenitor cells and improved the safety profile of the differentiation of myeloid, erythroid, and megakaryocytes. Additionally, HSK40495 has favorable PK properties and potent antitumor activity in BRCA knock-out (KO) colorectal cancer and BRCA-mutated breast cancer cell lines, and BRCA-mutated breast cancer CDX models [120].

DSB1559 demonstrated superior selectivity for PARP1 compared to AZD5305 in cell lines (Table 2). DSB1559 also showed highly desirable PK and ADME properties, indicating its high oral bioavailability in rodent PK studies. Compared with AZD5305, DSB1559 exhibited superior tumor residence time and tumor-to-plasma ratio and significantly reduced tumor growth while enhancing overall survival in a BRCA1-associated immune-competent TNBC model [121].

LAE119, a PARP1-selective inhibitor, demonstrated more than 1000-fold selectivity for PARP1 DNA trapping compared with PARP2, which has good PK profiles and ADME properties (Table 2). In a rat hematologic toxicity assay, LAE1 had minimal effects on hematological parameters and was well tolerated. LAE119 has more efficient anti-proliferative effects on BRCA1/2-mutated breast, colon, and pancreatic cancer cell lines than does AZD5305 and exhibits substantial tumor inhibition with reduced toxicity in BRCA-mutated breast and pancreatic cancer CDX models [122].

DHC-1, a potent and selective PARP1 inhibitor, selectively inhibited PARP1 activity and showed efficacy in BRCA1-deficient breast cancer and BRCA2-deficient pancreatic cancer cell lines (Table 2). DHC1 induced DNA damage and G2/M cell cycle arrest, resulting in enhanced sensitization to oxaliplatin. DHC-1 also exhibited a tumor-inhibitory effect in a CDX model of pancreatic cancer [123].

## Conclusions

PARPis altered the treatment paradigm in multiple cancers. PARPis not only improve the treatment outcome of patients with BRCA1/2-mutated cancers but also, as our understanding of HRD and BRCAness has increased, more patients may benefit from these therapies. PAOLA-1, a phase 3 clinical trial, supports potential benefit of HRD in improving the progression-free survival. This trial evaluated the therapy with Olaparib in newly diagnosed advanced ovarian cancer who were receiving chemotherapy and bevacizumab followed by bevacizumab. Prespecified subgroup analysis showed a beneficial progression-free survival in patients with BRCA-mutated and HRD-positive tumors. Furthermore, patients with HRD-positive tumors without BRCA mutation (20%) exhibited substantial clinical benefit compared to Olaparib [124]. Additional biomarkers for response to PARPi are being investigated in other solid tumors. As many cancer therapies may induce DNA damage and target DNA repair pathways, PARPis are potential partners for continually increasing the number of treatments with combination approaches. Even though the currently approved PARPis have shown significant clinical benefits, their toxicities continue to limit their use. Several next-generation PARPis exhibited improved PARP1 selectivity over PARP2, resulting in lower hematological toxicity while demonstrating greater therapeutic efficacy in multiple cancer models (Fig. 1C).

Currently, there is limited data on the resistance patterns for PARP1 inhibitors. Since HRR is most dependent on PARP1 rather than PARP2, we expect similar resistance mechanisms with possible PARP2 compensation. Larger studies will be instrumental in observing the resistance timelines, mechanisms, and patterns to PARP1 specific inhibitors.

Although more work is needed to determine the potential of these new inhibitors in the clinic, to establish drug resistance and effectiveness in combination therapy, the expanding landscape of preclinical research on the development of next-generation PARPis with increased PARP1 selectivity holds great promise for future options for patients. Furthermore, the improved toxicity profile of these new agents will allow for novel combinatorial strategies to further improve patient outcomes. Although there are several strategies for improving PARPi efficacy, increasing PARP1 selectivity is an exciting approach for improving efficacy and tolerability, which will lead to even wider adoption in future clinical practice.

### Key References

- Tung NM, Robson ME, Ventz S, Santa-Maria CA, Nanda R, Marcom PK, et al. TBCRC 048: Phase II Study of Olaparib for Metastatic Breast Cancer and Mutations in Homologous Recombination-Related Genes. J Clin Oncol. 2020;38(36):4274–82.
  - ○ Key clinical trial establishing the role of PARP inhibitors
- Turner NC, Balmana J, Poncet C, Goulioti T, Tryfonidis K, Honkoop AH, et al. Niraparib for Advanced Breast Cancer with Germline BRCA1 and BRCA2 Mutations: the EORTC 1307-BCG/BIG5-13/TESARO PR-30-50-10-C BRAVO Study. Clin Cancer Res. 2021;27(20):5482–91
  - ○ Key clinical trial establishing the role of PARP inhibitors
- Stodtmann S, Eckert D, Joshi R, Nuthalapati S, Ratajczak CK, Menon R, et al. Exposure–Response Model With Time-Varying Predictors to Estimate the Effects of Veliparib in Combination With Carboplatin/Paclitaxel and as Monotherapy: Veliparib Phase 3 Study in BRCA-Mutated Advanced Breast Cancer (BROCADE3) Trial. J Clin Pharmacol. 2022;62(10):1236–46
  - ○ Key trial in development of PARP inhibitors and combination strategies
- Dellavedova G, Decio A, Formenti L, Albertella MR, Wilson J, Staniszewska AD, et al. The PARP1 Inhibitor AZD5305 Impairs Ovarian Adenocarcinoma Progression and Visceral Metastases in Patient-derived Xenografts Alone and in Combination with Carboplatin. Cancer Res Commun. 2023;3(3):489–500
  - ○ Important work showing the promise of specific PARP1 inhibitors in ovarian cancer models
- Hopkins TA, Shi Y, Rodriguez LE, Solomon LR, Donawho CK, DiGiammarino EL, et al. Mechanistic Dissection of PARP1 Trapping and the Impact on In Vivo Tolerability and Efficacy of PARP Inhibitors. Mol Cancer Res. 2015;13(11):1465–77
  - ○ Seminal work defining the mechanisms of PARP inhibitor functions
- Farmer H, McCabe N, Lord CJ, Tutt AN, Johnson DA, Richardson TB, et al. Targeting the DNA repair defect in BRCA mutant cells as a therapeutic strategy. Nature. 2005;434(7035):917–21
  - ○ Defining BRCA mutation of a therapeutic biomarker
- Bryant HE, Schultz N, Thomas HD, Parker KM, Flower D, Lopez E, et al. Specific killing of BRCA2-deficient tumours with inhibitors of poly(ADP-ribose) polymerase. Nature. 2005;434(7035):913–7
  - ○ Defining BRCA mutation of a therapeutic biomarker
- Jamal K, Galbiati A, Armenia J, Illuzzi G, Hall J, Bentouati S, et al. Drug-gene Interaction Screens Coupled to Tumor Data Analyses Identify the Most Clinically Relevant Cancer Vulnerabilities Driving Sensitivity to PARP Inhibition. Cancer Res Commun. 2022;2(10):1244–54
  - ○ Establishing genomic vulnerabilities that sensitize cancer cells to PARP inhibitors

## Data Availability

No datasets were generated or analysed during the current study.

## Declarations

### Human and Animal Participants

This article does not contain any studies with human or animal subjects performed by any of the authors.

## Abbreviations

*PARP*: Poly ADP‒ribose polymerase
*SSB*: Single-strand break
*DSB*: Double-strand break
*BER*: Base-excision repair
*HRR*: Homologous recombination repair
*HRD*: Homologous recombination deficiency
*cNHEJ*: Classical nonhomologous end joining
*aNHEJ*: Alternative nonhomologous end joining
*I*: Inhibitor
*PK*: Pharmacokinetics
*PD*: Pharmacodynamics
*ADME*: Absorption, distribution, metabolism, and excretion
*BBB*: Blood–brain barrier
*GBM*: Glioblastoma
*TMZ*: Temozolamide
*TNBC*: Triple-negative breast cancer
*CDX*: Cell-derived xenograft
*PDX*: Patient-derived xenograft
